# Cardiovascular Health and Cognitive Function in Early Midlife: A Cross‐Sectional Analysis of the 1970 British Cohort Study

**DOI:** 10.1002/alz.084763

**Published:** 2025-01-09

**Authors:** Scott T Chiesa, Sarah‐Naomi James, Alun D Hughes

**Affiliations:** ^1^ MRC Unit for Lifelong Health and Ageing at UCL, London United Kingdom; ^2^ MRC Unit for Lifelong Health & Ageing at UCL, London United Kingdom

## Abstract

**Background:**

Poor cardiovascular health in midlife increases risk of dementia in later years. At least some of this risk may stem from early decrements in cognitive ability in those with poor cardiovascular health that are already evident by midlife. Whether such associations are causal, however, or develop in tandem due to shared factors encountered earlier in life, remains unclear.

**Method:**

A modified version of the American Heart Association Life’s Essential 8 (LE8) cardiovascular health score was constructed in 8,226 participants (age 47 years) recruited to the 1970 British Cohort Study. Four biological (BMI, blood pressure, glucose, total cholesterol) and four behavioral (diet, physical activity, smoking, sleep) metrics were each categorized as 0 (poor), 1 (intermediate), or 2 (ideal) and summed to create the LE8 score. A cognitive test battery assessing immediate and delayed memory recall, executive function, processing speed, and overall cognitive function were chosen as outcomes. Multiple linear regressions employing multiple imputation and adjusting for a range of early (childhood socioeconomic status, household overcrowding, childhood IQ, and highest education level) and mid‐life (current socioeconomic status and malaise) factors were used to conduct analyses whilst accounting for missing data.

**Result:**

Overall cognitive function in those classed as having ideal (LE8>13 points) cardiovascular health was 0.15 (0.04–0.27) z‐scores higher than those with intermediate (LE8 9‐13 points) and 0.42 (0.30–0.55) z‐scores higher than those with poor (<9 points) cardiovascular health. In age‐ and sex‐adjusted analyses, each 1‐point increase in LE8 was associated with a 0.02–0.06 z‐score higher cognitive function in all domains (p<0.001 for all). These associations became null for executive function and processing speed once accounting for early‐life factors, however, and were substantially attenuated for both memory domains. Further adjustment for midlife factors had little further impact on estimates. Follow‐up analyses suggested stronger relationships between midlife cardiovascular health behaviors and cognitive ability than biological factors.

**Conclusion:**

Much of the observed relationship between cardiovascular health and cognitive ability in early midlife is likely explained by other shared early‐life factors. While a modest association still persists after accounting for these factors, the directionality of this relationship remains to be determined.

